# Response to: Combined and Hybrid Treatments of Hyaluronic Acid (HA) and Calcium Hydroxylapatite (CaHA): A Systematic Review of Mechanisms of Action, Aesthetic Effectiveness, Satisfaction, and Safety Profile

**DOI:** 10.1007/s00266-025-05265-1

**Published:** 2025-10-08

**Authors:** Graeme Kerson, Andrew Schumacher

**Affiliations:** 1 Allergan Aesthetics, an AbbVie company, Marlow, UK; 2 Allergan Aesthetics, an AbbVie company, Irvine, CA USA

*Level of Evidence V* This journal requires that authors assign a level of evidence to each article. For a full description of these Evidence-Based Medicine ratings, please refer to the Table of Contents or the online Instructions to Authors www.springer.com/00266.

Dear Editor,

We would like to provide our critical insights and perspectives on the systematic review by Mecani et al., titled “Combined and Hybrid Treatments of Hyaluronic Acid (HA) and Calcium Hydroxylapatite (CaHA): A Systematic Review of Mechanisms of Action, Aesthetic Effectiveness, Satisfaction, and Safety Profile”^1^ published in the June 2025 issue of *Aesthetic Plastic Surgery*.

While the review analyzes distinct categories of administering HA and CaHA either as 2 separate treatments, hybrid syringe-to-syringe in-clinic mixed treatment, or hybrid manufactured ready-to-use treatment, the introduction describes a specific focus on the safety and effectiveness of off-label syringe-to-syringe in-clinic mixing of HA and CaHA. We appreciate the authors’ acknowledgment of licensed manufactured hybrid products that integrate both HA and CaHA into a single ready-to-use syringe that have obtained regulatory approvals due to their known safety profile and efficacy. In contrast, the practice of syringe-to-syringe in-clinic mixing currently lacks standardized validated protocols and any regulatory approvals. The authors make no justification for combining these treatment categories into one analysis; indeed, we would argue that they are distinct.

Analyzing each treatment protocol independently—as seen in similar reviews on hybrid fillers like Karaman-Zato 2024^2^—could enhance the clarity and depth of the analysis. In the Karaman-Zato review, the treatments are categorized into sequential (i.e., 2 separate treatments), hybridized in situ (i.e., in-clinic mixing), and pre-hybridized (i.e., licensed ready-to-use), and it discusses the off-label nature of in situ hybridization of HA and CaHA. In the Mecani et al. review, out of the 14 total human studies, 4 examine separate treatments, and only 3 address syringe-to-syringe in-clinic mixing of products. The remaining 7 studies use licensed manufactured ready-to-use hybrid products that already have well-established safety and efficacy profiles. Given this, the introduction should have reflected this broader scope to clearly inform the reader of the benefits and risks associated with both licensed and unlicensed syringe-to-syringe in-clinic mixed treatments.

Additionally, the Mecani et al. Table 1. Characteristics of the Human Studies excludes the study by Braz et al. (N = 403), which has a significant sample size and uses a licensed hybrid ready-to-use product. However, this study is later included in the overall safety conclusions (Table 6. Summary of Results on Safety and Adverse Effects), which would reinforce the safety of unlicensed syringe-to-syringe mixed products and, thus, misrepresent their perceived safety profile. Furthermore, the search criteria specified in the Supplementary Tables include 2 brand-name products from a single manufacturer. Considering the extensive range of available HA and CaHA products, expanding the scope to include a broader range of brand names or avoiding brand names altogether would enhance the analysis.

In summary, we believe the review’s conclusions extend beyond what can be supported by the methodological approach chosen. The analysis does not differentiate between licensed and unlicensed approaches and omits a broader product range inclusion. We urge readers to recognize the distinct profiles of licensed, ready-to-use products versus off-label, in-clinic mixed treatments.
